# Retraction: Genomic Instability in Liver Cells Caused by an LPS-Induced Bystander-Like Effect

**DOI:** 10.1371/journal.pone.0276208

**Published:** 2022-10-10

**Authors:** The PLOS ONE Editors

Following the publication of this article [1], concerns were raised regarding results presented in Figures 1, 2, 3, and 4. Specifically,

- The following immunohistochemistry results appear similar:
  - Figure 1A Control 4 Weeks and Figure 3A DNA 2 weeks when rotated 270°.
  - Figure 1A DNA 4 weeks, and Figure 3A DNA 4 week when rotated 270°.
  - Figure 1A LPS 2 weeks, and Figure 3A LPS 4 weeks when flipped along the vertical axis.
  - Figure 1A LPS 4 weeks, and Figure 3A LPS 2 weeks when flipped along the vertical axis.
  - Figure 1A Bacteria 4 weeks, and Figure 3A Bacteria 2 weeks when flipped along the horizontal axis and rotated 90°.
- The following western blot results appear similar:
  - Figure 4A Actin 4w lanes 1–10, Figure 4B DNMT3A 4w lanes 1–10, Figure 4D MeCP2 4w lanes 1–10, and Figure 2A Actin 2w lanes 1–10 when flipped along the vertical axis.
  - Figure 2A Actin 4w lanes 11–12 and Figure 4D MeCP2 4w lanes 11–12 when flipped along the vertical axis.
  - Figure 2A KU70 4w lanes 1–4, Figure 2A KU70 4w lanes 5–8, and Figure 4B Actin 2w lanes 5–8 when flipped along the vertical axis.
  - Figure 2B APE1 4w lanes 1–12, Figure 2C Actin 4w lanes 1–12, Figure 4B Actin 4w lanes 1–12, and Figure 4C Actin lanes 1–12.
  - Figure 2B Actin 4w lanes 1–12, Figure 2C Actin 2w lanes 1–12 when rotated 180°, and Figure 4D Actin 4w lanes 1–12.
  - Figure 2C PCNA 2w lanes 1–12 and Figure 4A DNMT1 4w lanes 1–12.
  - Figure 4A Actin 4w lanes 11–12 and Figure 4B DNMT3A 4w lanes 11–12.

The authors provided underlying data for the immunohistochemical results and the western blot data presented in this article, but these data did not resolve the concerns with the published figures. Following the discussion of these concerns with the authors, the corresponding author requested the retraction of this article.

IK, JT, and OK agreed with the retraction and apologized for the issues with the published article, but stand by the article’s findings. PW either did not respond directly or could not be reached.
